# Lagerstroemia speciosa Pers. (Lythraceae) Ethanolic Extract Attenuates Isoniazid-Induced Oxidative Stress and Hepatic Inflammation in Rats

**DOI:** 10.7759/cureus.51609

**Published:** 2024-01-03

**Authors:** T Rohit Singh, Devaraj Ezhilarasan, Munusamy Karthick, Karthik Shree Harini

**Affiliations:** 1 Pharmacology, Saveetha Dental College, Saveetha Institute of Medical and Technical Sciences, Saveetha University, Chennai, IND

**Keywords:** lagerstroemia speciosa, banaba, corosolic acid, drug induced liver injury, isoniazid

## Abstract

Background

Drug-induced liver injury is a common cause of acute liver failure. Isoniazid (INH) is used as a first-line treatment for tuberculosis. Clinical and experimental studies have reported abnormal liver function after INH therapy. *Lagerstroemia speciosa* Pers., commonly known as banaba, has been traditionally used to treat various ailments including diabetes and obesity due to its antioxidant and anti-inflammatory properties.

Aim

To investigate the hepatoprotective effect of ethanolic banaba leaf extract (EBLE) against INH-induced hepatotoxicity in rats.

Materials and methods

A total of 30 male Wistar albino rats (150 - 200 g) were divided into five groups (n = 6). Group I rats were served as a control and were administered dimethyl sulfoxide for the first 30 days and water for the next 30 consecutive days. Group II rats were administered INH (50 mg/kg, p.o.) once in the first 30 consecutive days and sacrificed at Day 30. Group III rats were administered INH for 30 consecutive days and left without treatment for the next 30 days. In Groups IV and V, rats were post-treated orally with EBLE 250 and 500 mg/kg, p.o. (0.3 ml/rat) for 30 days after INH administration. At the end of Day 60, the remaining group of animals were sacrificed. The blood and liver tissues were collected. The marker enzymes of hepatotoxicity, oxidative stress markers, inflammatory markers, and histopathology were analyzed.

Results

INH administration induced significant elevation of marker enzymes (aspartate transaminase, alanine transaminase, alkaline phosphatase, lactate dehydrogenase, bilirubin, gamma-glutamyl transpeptidase) of hepatotoxicity in the serum. This treatment also increased lipid peroxidation and proinflammatory marker expression (tumor necrosis factor-alpha, transforming growth factor-beta, and nuclear factor kappa B (NF-κB) except inhibitor of NF-κB) and decreased antioxidants such superoxide dismutase, catalase, and glutathione in the liver tissue. All these abnormalities were significantly mitigated after treatment with EBLE.

Conclusion

The results of this study suggest that EBLE can be used for INH-induced hepatotoxicity.

## Introduction

Drug-induced liver injury (DILI) is one of the major causes of acute liver failure (ALF). DILI is normally a dose-related phenomenon; however, it can also occur in the context of therapeutic doses. DILI occurs in a significant number of people exposed to drugs and generally is a challenging task for diagnosis and drug development [1]. More than a thousand drugs used in clinical practice are suspected to induce liver damage, of which 353 medications were convincingly related to liver injury [2].

DILI causes either apoptotic or necrotic liver cell death. The mechanisms of cell death and the severity of liver injury largely depend on the drug. Hepatic disruption accounts for 3.5-9.5% of all cases of adverse drug reactions and up to 14.7% of fatal adverse reactions. Liver diseases account for 3.5% of deaths globally. Given the population burden, India accounts for one-fifth (18.3%) of all cirrhosis deaths worldwide [3]. Particularly, there are certain drugs associated with hepatotoxicity that are prevalent in clinical practice. For instance, dapsone, acetaminophen, antiviral drugs, and anti-tubercular drugs like isoniazid (INH), rifampicin, and pyrazinamide cause hepatocellular degeneration [4].

Tuberculosis (TB), a major medical problem worldwide, continues to remain a major public health problem in developing countries, especially in India. INH and rifampicin are used as first-line drugs in the treatment of TB. However, consumption of INH doses of 20 mg/kg and >40 mg/kg are reported to cause acute and chronic toxicity in humans. Prolonged clinical treatment with INH is the most common cause of liver injury in patients receiving anti-TB therapy [4]. In the liver, N-acetyltransferase 2 enzyme metabolizes INH to acetyl INH, which subsequently hydrolyses into acetyl-hydrazine (AcHz), which is further oxidized by cytochrome P450 2E1 (CYP2E1) to form highly reactive acetylating hepatotoxic intermediates, causing hepatocellular degeneration [5]. Therefore, AcHz and hydrazine (Hz) derived metabolites were suggested to be responsible for INH-induced injury to the liver. The most characteristic features of serious INH-induced liver injury include hepatocellular injury with multilobular necrosis and mononuclear cell infiltration with early onset of steatosis. During biotransformation in the liver, INH induces reactive oxygen species (ROS) generation, which is associated with its hepatotoxic potential [6]. INH has been reported to interfere with pro-oxidant and antioxidant homeostasis, thereby stimulating the level of pro-oxidants or diminishing intracellular antioxidant levels by generating oxidative stress [7]. Hepatic disturbances due to DILI can alter homeostasis, which can lead to ALF. Therefore, there is an urgent need to identify the hepatoprotective principle that can be employed to combat INH-induced hepatotoxicity in the liver.

*Lagerstroemia speciosa *Pers. (Lythraceae), a tropical deciduous tree generally referred to as banaba, contains many polyphenolic compounds. These include ellagic acid and its derivatives, tannins, triterpenes, corosolic acid, flavones, leutins, quercetin, sitosterol, and glycosides [8, 9]. In our previous studies, we have reported the presence of berberin, ellagic acid, and gallic acids in ethanolic banaba leaf extract (EBLE) [10]. Tea derived from banaba leaves has historically been used in Southeast Asia to treat diabetes mellitus. Recent studies have reported the anti-diabetic, anti-inflammatory, antioxidant, antiviral, and antifibrotic potential of EBLE in animal studies [11]. Ethanolic extract of banaba petals was shown to possess antioxidant and free radical scavenging properties in carbon tetrachloride-induced liver injury in mice [12]. Our recent vitro studies have discussed the pro-apoptotic and cell cycle disrupting the ability for EBLE in liver cancer cells [10, 13]. The hepatoprotective efficacy of EBLE on INH-induced hepatotoxicity has not been studied. Therefore, in this study, EBLE was used to investigate the aspects of antioxidant and hepatoprotective effects against INH-induced hepatotoxicity in rats.

## Materials and methods

Drugs and plant extract

Isoniazid was purchased from Sigma chemicals (Hyderabad, India). EBLE was readily available and purchased from M/S. Quimico, herbal extract manufacturer, Bengaluru, India (Batch no. KAN/BE/1801009). According to the manufacturer’s certificate of analysis, EBLE contains 20% corosolic acid. The concentration of the extract was adjusted to 250 and 500 mg/kg for animal treatment. All the other chemicals used in this study were purchased from Sigma-Aldrich Company.

Experimental animals

Male Wistar rats (150-200 g) were used for the study. They were housed in clean polypropylene cages and maintained under standard laboratory conditions at a temperature of 22 ± 2°C and 12 hours with an alternating light-dark cycle. They were allowed free access to a standard pellet diet and water *ad libitum*. All the experimental procedures were conducted after obtaining necessary permission from the Institutional Animal Ethics Committee of Malla Reddy Institute of Medical Sciences, Hyderabad, India (3/IAEC/2017).

Experimental design

**Figure 1:**
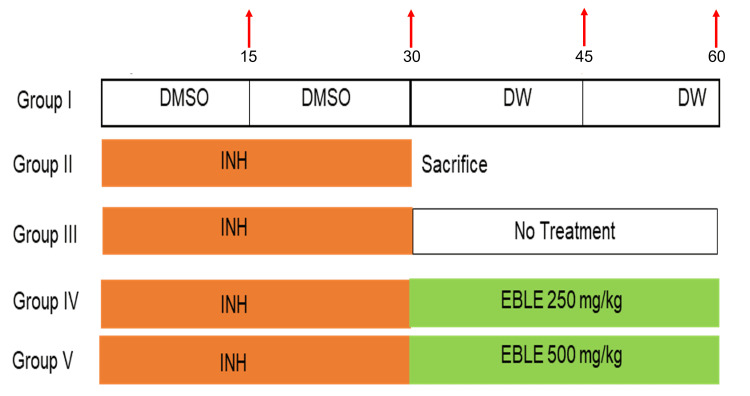
The schematic experimental design shows days of isoniazid and ethanolic banaba leaf extract administration in rats Red arrows indicate days of blood withdrawal. DMSO: dimethyl sulfoxide; DW: distilled water; INH: isoniazid; EBLE: ethanolic banaba leaves extract

Thirty animals were divided randomly into five groups (n = 6). Group I rats were administered dimethyl sulfoxide (DMSO) p.o. for the first 30 days and distilled water for the next 30 days and served as control. Group II rats were administered INH (50 mg/kg, p.o.) [14] once daily for 30 days and sacrificed on day 30th. Group III rats were administered INH, once daily for the first 30 days and were left without treatment for the next 30 days. Group IV and V rats received INH for 30 days once daily and were post-treated once daily with EBLE at a dose of 250 and 500 mg/kg b.w., p.o. (0.3 ml/rat) respectively [15] for the next 30 days. Except for Group II (sacrificed at the end of Day 30), all the animal groups were sacrificed at the end of Day 60 (Figure 1). INH was dissolved in DMSO, and EBLE was dissolved in distilled water.

The blood was collected at the end of Days 15, 30, 45, and 60 from overnight fasted rats to investigate the progression of hepatotoxicity by analyzing serum marker enzymes. The blood samples were centrifuged for 10 minutes at 1500 rpm and serum was separated and stored at -20°C until the analysis was carried out. At the end of the study, livers were excised, washed in ice-cold saline, blotted to dryness, and weighed. Liver tissues were homogenized with cold saline. After centrifugation at 3500 rpm for 10 minutes at 4°C, the supernatant was collected to measure superoxide dismutase (SOD), glutathione (GSH), and catalase (CAT) activities and malondialdehyde (MDA) content in the liver.

Estimation of serum marker enzymes of hepatotoxicity

Serum samples were used for the analysis of aspartate and alanine transaminases (AST and ALT), alkaline phosphatase (ALP), γ- glutamyl transpeptidase (GGT), lactate dehydrogenase (LDH) activities, and bilirubin content and were estimated according to the instructions of commercially available diagnostic kits (Enzo Life Sciences Inc., Farmingdale, NY).

Estimation of MDA

MDA content in liver tissue was determined by thiobarbituric acid (TBA) reaction as described by Okhawa et al. (1979) [16]. Liver tissue homogenate was mixed with 1.5 ml of 20% acetic acid, 0.2 ml of sodium dodecyl sulfate, and 1.5 ml of TBA was added in a tube and was made up to 4 ml with distilled water and then heated for 60 minutes at 95°C. After cooling, 4 ml of butanol-pyridine mixture was added and shaken well. After centrifugation at 4,000 rpm for 10 minutes, the organic layer was taken and its absorbance was measured at 532 nm.

SOD assay

To the tubes containing 0.5 ml of carbonate buffer, 0.5 ml of EDTA solution (the required amount of the homogenate-containing enzyme) was added and the final volume was made up to 2.5 ml. The reaction was initiated by the addition of 0.4 ml of epinephrine and the increase in absorbance at 480 nm was measured in a Shimadzu UV spectrophotometer (Shimadzu Corp., Kyoto, Japan). The enzyme activity was expressed as units/mg protein [17].

CAT assay

About 0.05 ml of tissue homogenate was added to 1.2 ml of the phosphate buffer. To this, 1 ml of hydrogen peroxide (H_2_O_2_) (0.2 M) was added to start the enzyme reaction. The decreased absorbance was taken at 620 nm for every 30-second interval for three minutes. The enzyme blank was used with 1 ml of distilled water. The catalase activity was measured in nM of H_2_O_2_ decomposed/min/mg protein [18].

Estimation of GSH

Reduced glutathione was assayed by the method of Moron et al. (1979) [19]. To 0.5 ml of tissue homogenate, 125 µl of 25% trichloroacetic acid (TCA) was added to precipitate proteins. The tubes were cooled in ice for five minutes and the mixture was further diluted with 0.6 ml of 5% TCA and centrifuged at 9000 RCF for 10 minutes. Then, 0.3 ml of the aliquot was made up to 1 ml with 0.2 M sodium phosphate buffer (pH 8.0) and freshly made 5,5-dithio-bis-(2-nitrobenzoic acid) reagent (2 ml). After 10 minutes of incubation, the intensity of the yellow color produced was measured using a spectrophotometer at 412 nm.

Gene expression analysis

TRIzol reagent was used to extract total RNA from liver tissue according to the manufacturer’s instructions (Invitrogen, ThermoFisher Scientific, Waltham, MA). The quality and quantity of total RNA were detected by a spectrophotometer (NANODROP200, ThermoFisher Scientific). The primers for tumor necrosis factor-alpha (TNF-α), transforming growth factor-beta (TGF-β), nuclear factor kappa B (NF-κB), and IκB (inhibitor of NF-κB) were synthesized from Sigma Genosys (Bangalore, India) (Table 1). The M-MuLV reverse transcriptase and RNase inhibitors were purchased from Thermo Scientific. PCR amplification was carried out on an Eppendorf Mastercycler® ep (Eppendorf AG, Hamburg, Germany). The PCR was done using initial denaturation at 94°C for one minute, 33 cycles of 94°C for 40 seconds, 65°C for 40 seconds, 72°C for 60 seconds, and a final extension at 72°C for two minutes. The amplified products were separated on a 1.5% agarose gel in tris buffer at 75V for three hours. The gel was stained with ethidium bromide and the amplified product was visualized and photographed on the gel documentation system. The location of a predicted product was confirmed by using a 100bp ladder (Applied Biosystems, ThermoFisher Scientific) as a standard-size marker. The gel was then photographed under UV transillumination. The intensity of PCR products was measured using a video image analysis system (Kodak Digital Science, Rochester, NY). The signal for each transcript was standardized against that of the β-actin mRNA from each sample and the results were expressed as transcript/β-actin mRNA ratio.

**Table 1:** Primers used in this study NF-κB: nuclear factor kappa B; TNF-α: tumor necrosis factor α; TGF-β: transforming growth factor β; IκB: inhibitor of NF-κB.

Oligo Name	5' 3'	Base pairs
NF-kB	Forward:5′-GCAGATGGCCCATACCTTCA-3′	123
	Reverse: 5′-CACCATGTCCTTGGGTCCAG-3′	
β-actin	Forward: 5´-GCTCGTCGTCGACAACGGCTC-3′	480
	Reverse: 5´-CAAACATGATCTGGGTCATC-3′	
TNF-α	Forward: 5′-GCCTCTTCTCATTCCTGCTTG-3′	296
	Reverse: 5′-CTGATGAGAGGGAGGCCATT-3′	
β-actin	Forward: 5´- TCTTCCAGCCATCCTTCTTG -3´	108
	Reverse: 5´- CGGTGATTTCCTTCTGCATT -3´	
TGF-β	Forward: 5′-CCGATGGGTTGTACCTTGTC -3′	525
	Reverse: 5′-GGGCTGGGTAGAGAATGGAT -3′	
β-actin	Forward: 5´- TCATGAAGTGTGACGTTGACA -3´	696
	Reverse: 5´- CCTAGA AGCATTTGCGGTGCA -3´	
IκB	Forward: 5’-TGGCTCATCGTAGGGAGTTT-3′	360
	Reverse: 5’-CTCGTCCTCGACTGAGAAGC-3′	
β-actin	Forward: 5’-CGTGGGCCGCCCTAGGCACCA-3′	638
	Reverse: 5’-TTGGCCTTAGGGTTCAGGGGG-3′	

Histopathology of liver tissue

The liver tissue was fixed in 10% formalin, dehydrated in gradual ethanol (50-100%), cleared in xylene, and embedded in paraffin wax. The wax sections with 5-6 micron thickness were made using a rotary microtome. These tissue sections were then stained with hematoxylin and eosin, Masson’s trichrome, and Sudan black B [20].

Statistical analysis

Data obtained from the experiments are expressed as mean ± SD for six animals. The data were subjected to one-way analysis of variance (ANOVA); post hoc multiple comparison tests to assess the degree of significance of the difference between means of various treatment groups were performed by employing Tukey’s test, using SPSS software v. 22.0 (IBM Corp., Armonk, NY). The p-value < 0.05 was considered significant.

## Results

Effect of EBLE on INH-induced changes in hepatotoxic marker enzymes and bilirubin in serum

**Figure 2:**
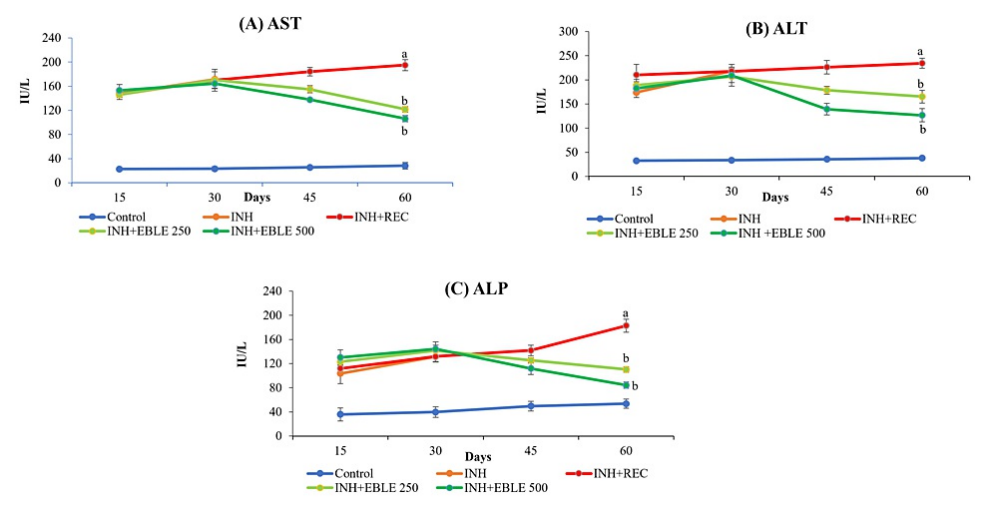
Effect of EBLE on INH-induced changes seen in the marker enzymes of hepatotoxicity (AST ALT, and ALP) Reduced levels of AST, ALT, and ALP in the serum of rats upon EBLE treatment. INH and INH+REC group rats were sacrificed on Days 30 and 60, respectively. ^a^*p* < 0.001 vs control; ^b^*p* < 0.001 vs INH+REC. EBLE: ethanolic banaba leaves extract, INH: isoniazid: REC: recovery; AST: aspartate transaminase; ALT: alanine transaminase; ALP: alkaline phosphatase

**Figure 3:**
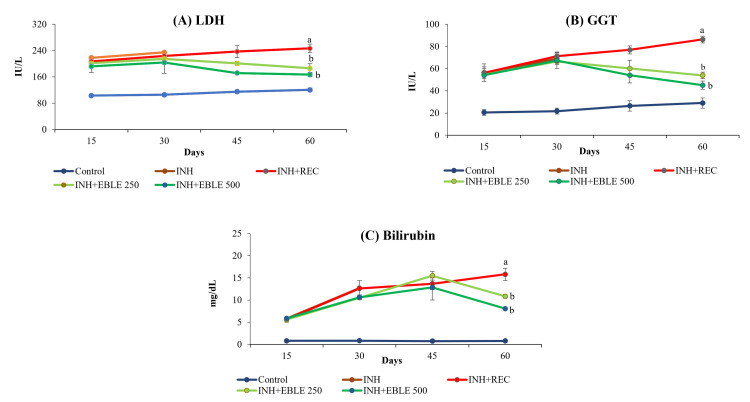
The effect of EBLE on INH-induced changes seen in the marker enzymes of hepatotoxicity (LDH, GGT, and bilirubin) Reduced levels of LDH, GGT, and bilirubin in the serum of rats upon treatment with EBLE. INH and INH+REC group rats were sacrificed on Days 30 and 60, respectively. ^a^p < 0.001 vs control; ^b^p < 0.001 vs INH+REC. EBLE: ethanolic banaba leaves extract, INH: isoniazid: REC: recovery; LDH: lactate dehydrogenase; GGT: gamma-glutamyl transpeptidase

In Group II rats, 30 days of INH administration caused significant (p< 0.001) elevation of serum marker enzymes of hepatotoxicity such as AST, ALT, ALP, LDH GGT, and bilirubin as compared to the Group I control rats. Interestingly, INH administration caused a nearly three-to-fourfold increase in liver marker enzymes, as measured at the end of Days 15 and 30. The hepatotoxic potential of Group II rats (sacrificed at the end of 30 days) after INH administration was similar to that of Group III (left without treatment after 30 days of INH administration) and hence, INH + EBLE 250 mg/kg (Group IV) and INH + EBLE 500 mg/kg (Group V) post-treated groups were compared to Group III. As compared to Group III rats, EBLE treatments for 30 days after INH administration caused a significant fall (p < 0.001) in the elevation of all of the above marker enzymes and bilirubin in the serum (Figures 2-3).

Effect of EBLE on INH-induced changes in lipid peroxidation and antioxidants in liver tissue of rats

In Groups II and III rats, INH administration for 30 days caused a significant (p < 0.001) increase in liver MDA level as compared to the Group I control rats. INH treatments also caused a significant (p < 0.001) decrease in SOD and CAT activities and GSH content in the liver tissue of rats (Group II and III). EBLE administered at doses of 250 mg/kg and 500 mg/kg as post-treatments for 30 days after INH administration significantly reduced MDA levels (p < 0.001) and replenished GSH content in liver tissue as compared to Group III rats. The EBLE administration at 250 mg/kg mildly reversed SOD activity (p < 0.05), whereas EBLE at a dose of 500 mg/kg significantly reversed SOD activity (p < 0.01). The EBLE post-treatment at a dose of 500 mg/kg replenished CAT and GSH highly significantly to near normalcy (p < 0.001) and also significantly decreased INH-induced CAT activity in liver tissue at a dose 250 mg/kg (p < 0.01) (Table 2).

**Table 2:** Effect of EBLE against INH-induced changes in the status of enzymic and non-enzymic antioxidants in the liver tissue of rats Values presented are mean ± S.D. for ‘n’ no. of animals mentioned in parenthesis. Group I: Control; Group II: INH alone treated rats; Group III: INH+ Recovery; Group IV: INH+EBLE 250 mg/kg; Group V: INH+EBLE 500 mg/kg. INH and INH+REC group rats were sacrificed on Days 15 and 30, respectively. Multiple comparison between treatment groups was performed by Tukey’s test. arefers to the control group compared to INH and INH+REC; b refers to the INH+REC group compared to INH+EBLE 250 mg and INH+EBLE 500 mg; *p < 0.05, **p < 0.01, ***p < 0.001. LPO: lipid peroxidation; SOD: superoxide dismutase; CAT: catalase; GSH: reduced glutathione

Treatment Groups (n=6)	Parameters			
	LPO	SOD	CAT	GSH
	(nmol/mg protein)	(Units/mg protein)	(Units/mg protein)	(Units/mg protein)
Group I	3.26 + 0.30	10.58 + 0.55	7.50 + 0.43	7.43 + 0.50
Group II	9.33 + 0.62 a***	4.05 + 0.55 a***	2.62 + 0.67 a***	2.86 + 0.13 a***
Group III	10.79 + 0.60 a***	3.96 + 0.09 a***	2.27 + 0.67 a***	1.47 + 0.43 a***
Group IV	8.80 + 0.46 b***	4.86 + 0.61 b*	3.56 + 0.32 b**	2.90 + 0.34 b***
Group V	6.81 + 0.41 b***	5.21 + 0.33 b**	4.36 + 0.24 b***	4.02 + 0.61 b***

Effect of EBLE on INH-induced changes in the pro-inflammatory marker genes expressions in liver tissue

**Figure 4:**
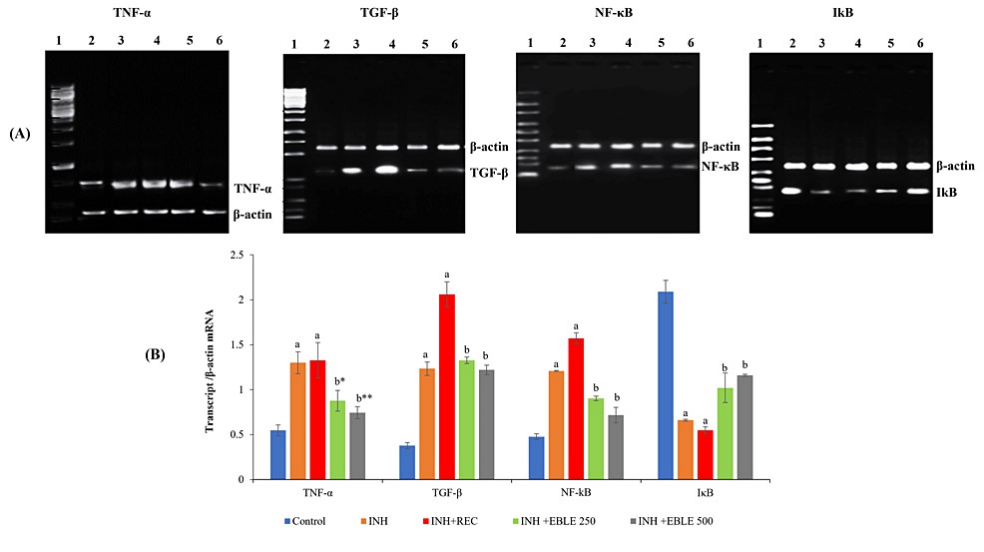
Effect of EBLE on INH-induced changes in the expression of inflammatory and fibrosis genes Reduced level of inflammatory and fibrosis genes in the EBLE treatment on INH-induced rats of liver tissue. A: Qualitative expression of the above genes: 1-Marker; 2-Control; 3-INH; 4-INH+REC; 5 INH+EBLE 250 mg/kg; 6-INH+EBLE 500 mg/kg. B: Quantitative analysis of gene expression: ^a^*p* < 0.001 vs control; ^b*^p < 0.05; ^b**^p < 0.01; ^b^*p* < 0.001 vs INH+REC group TNF: α-tumor necrosis factor-α; TGF-β: transforming growth factor-β; NF-κB: nuclear factor kappa B; IκB: inhibitor of NF-κB

In this study, INH administration for 30 days caused significant (p < 0.001) upregulation of pro-inflammatory gene expressions such as TNF-α, TGF-β, and NF-κB and downregulation of IκB gene expression in the liver tissue of rats. After INH administration for 30 days, EBLE post-treatment with 250 mg/kg (p < 0.05) and 500 mg/kg (p < 0.01) doses caused significant downregulation of TNF-α gene expression. EBLE post-treatment at doses of 250 mg/kg and 500 mg/kg caused significant (p < 0.001) downregulation of TGF-β and NF-κB gene expressions with a concomitant upregulation (p < 0.001) of IκB gene expression (Figure 4).

Effect of EBLE on INH-induced histopathological changes in liver tissue

The liver architectural distortion was investigated by H and E staining. The control liver tissues showed a normal architecture of the liver with an intact central vein. The liver tissue of INH-administered rats (Group II) showed degeneration of hepatocytes along with sinusoidal dilatation. The liver tissues of Group III rats showed sinusoidal dilatation along with hepatocyte degeneration and centrilobular necrosis with infiltration of inflammatory cells. The liver tissues of Group IV rats post-treated with EBLE 250 mg/kg showed mild periportal inflammation. The liver tissues of Group V rats post-treated with EBLE 500 mg/kg showed a normal portal vein and normal hepatocytes.

Masson's trichrome staining was performed to assess the fibrotic changes in the liver tissue. Control rat liver tissue showed normal liver architecture with no extracellular matrix accumulation. Group II and III rats' liver tissues showed mild peribiliary fibrosis (PBF) with sinusoidal dilatation. The liver tissue of Groups IV and V rats, which were post-treated with EBLE 250 mg/kg and 500 mg/kg dose showed moderate PBF and mild PBF with mild inflammation respectively.

**Figure 5:**
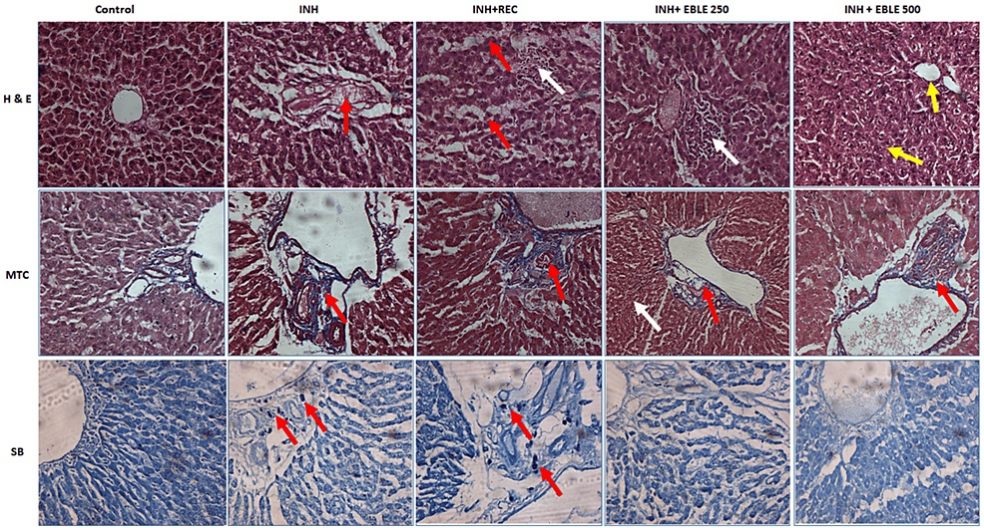
Histopathology of the liver Hematoxylin and eosin (H & E) staining (20x), scale bar-50µm The control shows the normal liver architecture. In the isoniazid (INH) alone group, the foci of hepatocyte degeneration and sinusoidal dilatation were observed. In the INH+REC group, sinusoidal dilatation with degeneration (red arrow) and foci of centrilobular necrosis with infiltration of inflammatory cells (white arrow) were observed; in INH+EBLE 250 mg/kg group, the foci of periportal inflammation was observed; in INH+EBLE 500 mg/kg group the rat liver tissue shows normal portal vein and normal hepatocytes (yellow arrow). Masson’s trichrome staining (MTC) The control rat's liver tissue shows normal liver architecture, and the INH alone group shows mild peribiliary fibrosis (PBF). INH+REC group shows moderate PBF with sinusoidal dilatation, in the INH+EBLE 250 and 500 mg/kg groups, the white arrow shows normal hepatocytes, and the red arrow shows moderate and mild peribiliary inflammation with fibrosis. Sudan Black (SB) staining Control shows no appearance of black color fat droplets. The INH and INH+REC groups show black color fat droplets. The liver architecture of the INH+EBLE group does not contain a black stain of lipid droplets.

After Sudan black staining, the control liver tissue showed no appearance of black color fat droplets. Group II and Group III rat liver tissues showed the appearance of black color fat droplets. The liver tissue of Groups IV and V rats which were post-treated with EBLE (250 mg/kg and 500 mg/kg) did not show a black stain of lipid droplets (Figure 5).

## Discussion

Clinical studies in the past revealed that the cure and date rates of patients treated with anti-TB drugs were 11.42% and 40.9%, respectively. However, when the patients were administered anti-TB drugs (e.g. INH) along with plant formulations such as silymarin and curcumin, the cure and death rates were 41.3% and 3.8%, respectively [21]. Clinically, INH-induced liver toxicity is usually asymptomatic. Measuring serum markers of hepatocyte injury such as AST, ALT, and ALP indicate the onset of hepatic damage. During hepatocellular degeneration, injured hepatocytes release these intracellular enzymes in the blood that cause elevation of liver transaminases (AST and ALT), ALP, LDH, and GGT [22]. INH metabolism in the liver by CYP2E1 causes the liberation of excessive intracellular free radical accumulation. Increased intracellular ROS is responsible for oxidative stress in hepatocytes and thus plays an important role in INH-induced hepatotoxicity. Liberation of ROS and other free radicals are associated with the process of LPO, oxidative stress, and loss of membrane integrity, which is collectively responsible for liver injury [23]. In light of these reports, it is likely that INH-induced oxidative damage may result in the acceleration of LPO and cause injury to the hepatocyte membrane, and this could be the most probable cause for the INH-induced elevation of marker enzymes of hepatotoxicity in serum. Many plant extracts and their derived phytochemicals have been studied to evaluate their efficacy versus anti-TB drug-induced hepatic damage in different animal models [11].

The hepatoprotective effect of those herbal extracts was mainly attributed to the hepatocellular membrane stabilizing properties due to the presence of several phytochemicals. These phytochemicals are implicated in the restoration of membrane stability and recovery from hepatic damage [11]. In our previous studies [10, 13], we have reported the presence of berberine, gallic acid, and ellagic acid in EBLE. Therefore, the fall in liver marker enzymes in serum after EBLE treatments can be attributed to the membrane-stabilizing properties of the above phytochemicals.

The metabolites of INH act as initiators of lipid peroxidation that cause hepatic necrosis. In this study, INH administration induced lipid peroxidation and concomitantly decreased first-line antioxidants such as SOD, CAT, and GSH in the liver tissue of rats indicating the onset of oxidative stress. SOD scavenges superoxide anions and forms H_2_O_2_. CAT is an enzymatic antioxidant and it degrades H_2_O_2_ to protect tissues from hydroxyl radicals. Therefore, a decrease in SOD activity is a sensitive indicator of oxidative stress-induced hepatic injury. An imbalance between the overall pro-oxidant and antioxidant activity leads to oxidative stress. During oxidative stress, the antioxidant enzymes were produced in large amounts to scavenge the free radicals [24]. Previous studies also reported the diminution of GSH and decreased glutathione-S-transferase, CAT, and SOD activities after INH administration induced oxidative stress in rats [25]. The increased LPO upon INH administration could be due to the liberation of Achz and Hz and other free radicals after its metabolism by CYP2E1. The decreased first-line antioxidant defense upon INH administration could be due to the overutilization of these enzymes against free radicals liberated by INH metabolism. Phytochemicals are effective in the management of INH-induced toxicity as they mainly act on cytochrome P450 and reduce INH-induced free radicals [20]. Previous studies have also used banaba extract against oxidative stress-induced hepatotoxicity, in which banaba extract administration was shown to offer hepatoprotective activity in CCl_4_-induced hepatotoxicity [13]. EBLE has also been reported to possess potent antioxidants, free radical scavenging activities, and hepatocellular membrane-stabilizing properties due to the presence of triterpenoids and sterols [14]. Therefore, the presence of triterpenoids, such as corosolic acid, could be attributed to the antioxidant activity of EBLE.

TNF-α is an inflammatory cytokine produced by macrophages that regulates macrophage function and plays a central role in inflammatory cell activation and hepatic inflammation. TNF-α up-reregulation also induces NF-κB activation-related signaling transduction pathways during inflammation. NF-κB is involved in the pro-inflammatory signaling pathway, which is activated by several proinflammatory cytokines. It has been reported that the activation of NF-κB plays an important role in the initiation and progression of INH-induced liver damage [26]. NF-κB is composed of dimers, which are inactive in the cytoplasm, and it binds to IκB, which is an inhibitory protein. The inhibitory protein IκB on stimulation is phosphorylated and degraded by IκB kinase, and NF-κB is released and transferred to the nucleus and triggers the release of proinflammatory mediators like TNF-α [27]. TGF-β signaling plays an important role in cell damage; oxidative stress, liver fibrosis, and enhanced TNF-α, TGF-β, and NF-κB expressions were often reported as the primary cause for various hepatotoxic drug-induced liver injuries [28]. In this study, INH-induced pro-inflammatory marker gene expressions such as TNF-α, TGF-β, and NF-κB are responsible for liver inflammation and fibrosis and this could be the possible cause for the onset of INH-induced liver inflammation and hepatotoxicity. Corosolic acid from banaba has been shown to ameliorate acute inflammation via inhibition of interleukin-1 receptor-associated kinase 1 phosphorylation in macrophages [29]. Ethanolic extract of banaba has been shown to possess an anti-inflammatory effect against carrageenan-induced acute inflammation and formalin-induced (chronic) paw edema models [30]. Therefore, the presence of triterpenoids such as corosolic acid in EBLE can be attributed to its anti-inflammatory potential [14].

**Figure 6:**
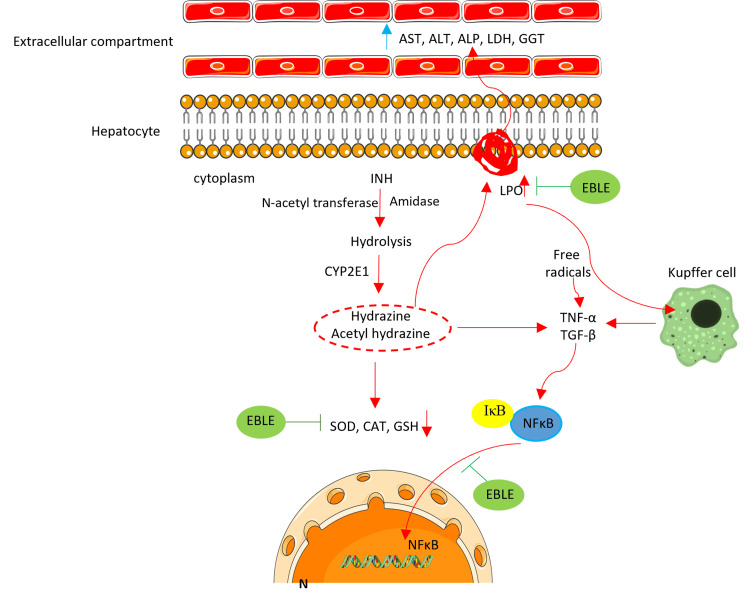
Mechanism of isoniazid (INH)-induced hepatotoxicity and hepatoprotective effects of ethanolic banaba leaves extract (EBLE) in rats AST and ALT: aspartate transaminases and alanine transaminases; ALP: alkaline phosphatase, LDH: lactate dehydrogenase, GGT-γ glutamyl transpeptidase; LPO: lipid peroxidation; SOD: superoxide dismutase; CAT: catalase; GSH: reduced glutathione; TNF-α: tumor necrosis factor-alpha; TGF-β: transforming growth factor-beta; NF-κB: nuclear factor kappa B; IκB: inhibitor of NF-κB; N: nucleus Image credits: Devaraj Ezhilarasan

In the present study, histopathology of the liver also confirms that INH can induce architectural distortion and fibrotic changes along with degeneration of hepatocytes and inflammation. EBLE post-treatments, especially at 500 mg/kg, significantly reduce INH-induced degenerative changes and fibrosis in the liver. Our biochemical results are well-corroborated with pathological and molecular analysis. The probable hepatoprotective effect of EBLE is presented in Figure 6.

Limitations

This study reports the hepatoprotective effect of banaba on INH-induced liver injury. INH is also known to induce liver steatosis, and this study has not focused on the hepatic steatosis aspects. Future studies are required to investigate the influence of banaba on INH-induced changes in lipid metabolism and lipotoxicity. Future studies should explore the metabolic interaction of banaba extract-based phytochemicals with INH metabolites and its possible role in the prevention of hepatotoxicity.

## Conclusions

In conclusion, the present study suggests that once daily, 30 days of INH administration induces significant hepatotoxicity in rats. The injured liver did not recover from INH intoxication even after 30 days left without any treatment and the toxicity was similar to the INH alone group (Group II), which was sacrificed at the end of Day 30. At a "high" dose, EBLE post-treatment significantly reduced INH-induced hepatotoxicity, and hence, EBLE could be further studied to develop as a potential hepatoprotective drug against INH-induced liver toxicity.
